# Response to mepolizumab treatment is sustained across 4-weekly dosing periods

**DOI:** 10.1183/23120541.00068-2020

**Published:** 2020-09-14

**Authors:** Ian D. Pavord, Eugene R. Bleecker, Roland Buhl, Pascal Chanez, Elisabeth H. Bel, Peter Howarth, Daniel J. Bratton, Frank C. Albers, Steven Yancey

**Affiliations:** 1 Respiratory Medicine Unit and Oxford Respiratory NIHR BRC, Nuffield Dept of Medicine, University of Oxford, Oxford, UK; 2 Genomics and Precision Medicine, University of Arizona College of Medicine, Tucson, AZ, USA; 3 Dept of Pulmonary Medicine, Mainz University Hospital, Mainz, Germany; 4 Clinique des bronches allergies et sommeil, INSERM C2VN Center and CIC Nord APHM, Aix-Marseille University, Marseille, France; 5 Dept of Respiratory Medicine, Amsterdam University Medical Center, University of Amsterdam, Amsterdam, The Netherlands; 6 Global Medical Franchise, GSK, Brentford, UK; 7 Clinical Statistics, GSK, Stockley Park, UK; 8 Respiratory Medical Franchise, GSK, Research Triangle Park, NC, USA; 9 Respiratory Therapeutic Area, GSK, Research Triangle Park, NC, USA

## Abstract

**Background:**

Mepolizumab (100 mg delivered *s.c.* every 4 weeks) is indicated for add-on maintenance treatment for patients with severe eosinophilic asthma. Mepolizumab has been shown to reduce exacerbations and the requirement for daily oral corticosteroids, and improve asthma control and symptoms. However, data on the durability of the response to mepolizumab during dosing periods are limited. The aim of this study was to investigate the efficacy profile in patients with severe eosinophilic asthma over the 4-weekly dosing period for various fixed mepolizumab doses.

**Methods:**

This was a *post hoc* analysis of data from the phase IIb/III DREAM study. Patients ≥12 years of age with severe eosinophilic asthma were randomised (1:1:1:1) to receive intravenous mepolizumab 75 mg (equivalent to 100 mg *s.c.),* 250 mg, 750 mg or placebo, plus standard of care, every 4 weeks for 52 weeks. The number of exacerbations and eDiary data (peak expiratory flow, rescue medication use and symptom scores) from two periods in each 4-weekly dosing interval (days 1–14 and 15–28) over the 52-week treatment period were analysed.

**Findings:**

eDiary data and the proportion of patients experiencing ≥1 exacerbation were similar during the first and second 2 weeks of a dosing period across all mepolizumab doses.

**Interpretation:**

These results demonstrate that the response to mepolizumab is sustained over the 4-weekly dosing period with no differences across a 10-fold dose range and supports the use of the current mepolizumab dosing regimen in patients with severe eosinophilic asthma.

## Abstract 2

***Post hoc* analysis of data from DREAM demonstrated a sustained response to mepolizumab across the 4-weekly dosing period, suggesting therapeutic benefit is maintained between each mepolizumab dose following long-term treatment** https://bit.ly/3843lH6

## Introduction

Asthma is a heterogeneous disease consisting of several inflammatory sub-phenotypes, one of which is severe eosinophilic asthma [1, 2]. Patients with severe eosinophilic asthma show persistent eosinophilic airway inflammation, often experience frequent exacerbations and have an impaired quality of life, despite receiving treatment with high-dose inhaled corticosteroids (ICSs) and/or oral corticosteroids (OCSs) and other controllers [1, 3]. One of the current treatment options for patients with severe eosinophilic asthma is mepolizumab, an anti-interleukin-5 monoclonal antibody that selectively inhibits eosinophilic inflammation [4]. Mepolizumab is approved as an add-on maintenance treatment to standard of care, and the current mepolizumab dosing regimen involves *s.c.* administration of a fixed dose (100 mg) once every 4 weeks [5, 6].

Clinical trials have demonstrated that mepolizumab treatment reduces blood eosinophil counts, the rate of clinically significant exacerbations, and the requirement for daily OCS use, and improves lung function, asthma control, and health-related quality of life compared with placebo in patients with severe eosinophilic asthma [7–10]. Specifically, the phase IIb/III DREAM (Dose Ranging Efficacy And safety with Mepolizumab in severe asthma) study demonstrated that mepolizumab treatment with either 75 mg (bioequivalent to 100 mg *s.c*.) [11], 250 mg, or 750 mg (intravenous (*i.v*.) administration) was associated with reductions in the rate of exacerbations and blood eosinophil counts, with minimal differences seen across the three treatment groups [9]. Data from the open-label extension study, COLUMBA also showed that clinical and pharmacodynamic responses to mepolizumab treatment were sustained over 4.5 years in patients with severe eosinophilic asthma, with only a small proportion of patients (3%) withdrawn from the study due to a lack of treatment efficacy [12]. However, none of the previous clinical studies of mepolizumab have investigated whether treatment response is sustained or decreases between mepolizumab doses, and whether the mepolizumab dose and patient characteristics influence this. These are important questions as a waning of treatment response might suggest inadequate dosing.

The aim of this *post hoc* analysis was to evaluate the efficacy profile of mepolizumab over the 4-weekly dosing period for various fixed doses of mepolizumab (75 mg, 250 mg, 750 mg *i.v*.) in patients with severe eosinophilic asthma using data from the DREAM study.

## Materials and methods

### Study design

This was a *post hoc* analysis of data from the multicentre, randomised, double-blind, placebo-controlled, parallel group study, DREAM (GSK ID: MEA112997; ClinicalTrials.gov identifier: NCT01000506) [9]. The design of this study has been reported previously [9]. In brief, patients with severe eosinophilic asthma were randomly assigned 1:1:1:1 to receive mepolizumab 75 mg, 250 mg, 750 mg *i.v*., or placebo, in addition to standard of care, every 4 weeks for 52 weeks. The DREAM study was conducted in accordance with the ethical principles of the Declaration of Helsinki, International Conference on Harmonization Good Clinical Practice Guidelines, and applicable country-specific regulatory requirements.

### Patients

Eligible patients included those ≥12 years of age with severe eosinophilic asthma and a history of ≥2 exacerbations requiring treatment with systemic corticosteroids in the year prior to enrolment despite receiving regular treatment. In addition, these patients required treatment with high-dose ICSs in the 12 months before screening and additional controller therapy with or without maintenance OCSs. Further details on eligibility criteria have been reported previously [9]. Severe eosinophilic asthma was defined as a blood eosinophil count of ≥300 cells·µL^−1^, a sputum eosinophil count of ≥3%, fractional exhaled nitric oxide of ≥50 ppb, or a deterioration of asthma control following a ≤25% reduction in regular-maintenance ICSs or OCSs in the previous year. Data from the intent-to-treat (ITT) population in DREAM who had been randomised and received ≥1 dose of study treatment were included in this analysis.

### End-points and assessments

Patients were asked to record the following parameters daily in an electronic diary (eDiary) from the first visit onwards: morning peak expiratory flow (best of three) before rescue medication usage (L·min^−1^); occasions of rescue medication (short-acting β_2_-agonists) usage over the previous 24 h; asthma symptom score over the previous 24 h. Peak expiratory flow was measured using an electronic peak flow meter (Piko Peak Flow Meter) that transmitted the peak flow value to the eDiary (eSense). The asthma symptom score was assessed using the following scale: 0: no symptoms during the previous 24 h; 1: symptoms for one short period during the previous 24 h; 2: symptoms for two or more short periods during the previous 24 h; 3: symptoms for most of the previous 24 h that did not affect my normal daily activities; 4: symptoms for most of the previous 24 h that did affect my normal daily activities; and 5: symptoms so severe that I could not go to work/school or perform normal daily activities.

Clinically significant exacerbations were corroborated by changes from baseline in ≥1 of the following parameters recorded in a patient's eDiary: a decrease in peak flow, an increase in the use of rescue medication, an increase in the frequency of nocturnal awakening due to asthma symptoms, or an increase in overall asthma symptom score. Clinically significant exacerbations were defined as worsening of asthma requiring use of OCSs for ≥3 days and/or hospitalisation and/or an emergency department visit. For patients receiving maintenance OCSs, an exacerbation requiring OCSs was defined as the use of oral/systemic corticosteroids at least double the existing maintenance dose for ≥3 days.

### Sample size and statistical analysis

All analyses were performed descriptively. For the main analysis, data on exacerbations and eDiary data (peak expiratory flow, rescue medication use, and symptom score) were averaged across all 4-weekly dosing periods from the 52-week study, and treatment responses compared between the first 2 weeks after mepolizumab administration (days 1–14) and the second 2 weeks after mepolizumab administration (days 15–28). The analysis of each dosing period included patients with ≥7 days of eDiary data in each of the 2-week periods. If doses were less than 28 days apart, the midpoint between doses was used to divide the eDiary data into periods of equal size. In addition, treatment responses for peak expiratory flow, rescue medication use, and symptom score were compared for the first 2 weeks and second 2 weeks of each individual 4-weekly dosing period (weeks 0–4 to 48–52). Owing to the small number of exacerbation events recorded during each 4-weekly dosing period, only data combined across all 4-weekly dosing periods were used for the exacerbation end-point.

## Results

### Patient population

This analysis included all patients from the DREAM ITT population (n=616). As reported previously, baseline demographics were similar across the treatment groups [9]. In addition, baseline clinical characteristics such as blood eosinophil counts, percentage of predicted prebronchodilator forced expiratory volume in 1 s, and exacerbations requiring admission in the previous year were similar between the treatment groups [9].

### Clinical efficacy of mepolizumab during the first 2 weeks (days 1–14) and second 2 weeks (days 15–28)

**FIGURE 1:**
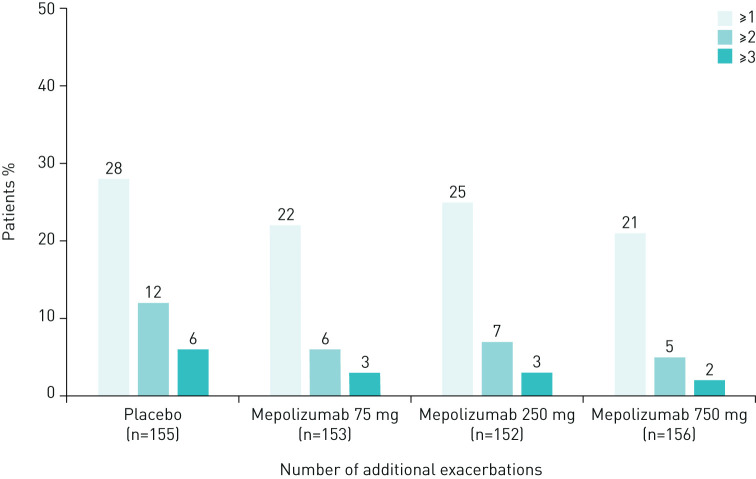
Proportion of patients with ≥1, ≥2 and ≥3 additional exacerbations during the second 2 weeks compared with the first 2 weeks (averaged across all 4-weekly dosing periods within the DREAM study). Excludes 33 exacerbations (4% of all exacerbations: 31 occurred more than 28 days after the most recent dose; 2 occurred on the day of the first dose).

The proportion of patients experiencing ≥1 exacerbation was similar during the first and second 2 weeks of a dosing period, averaged across all 4-weekly dosing periods (table 1). Similar results were observed across all treatment groups and, as expected, during both dosing periods the total number of exacerbations was lower in the mepolizumab treatment groups compared with placebo (table 1). In addition, the proportion of patients experiencing ≥1, ≥2, and ≥3 additional exacerbations during the second 2 weeks compared with the first 2 weeks of a dosing period was found to be numerically greater for the placebo group compared with the three mepolizumab treatment groups (figure 1; table 1). When data were analysed according to baseline blood eosinophil count, there was no obvious difference between patients with <300 cells·µL^−1^ *versus* those with ≥300 cells·µL^−1^ across all mepolizumab treatment groups (supplementary table 1).

**TABLE 1:** Number of exacerbations during dosing periods (averaged across all 4-weekly dosing periods within the DREAM study)

**Dosing period**	**Placebo (n=155)**	**Mepolizumab**		
		**75 mg (n=153)**	**250 mg (n=152)**	**750 mg (n=156)**
**First 2 weeks (days 1–14)**				
Total exacerbations n	137	67	84	69
Mean exacerbations per patient n	0.88	0.44	0.55	0.44
≥1 exacerbation n (%)	75 (48)	44 (29)	52 (34)	46 (29)
**Second 2 weeks (days 15–28)**				
Total exacerbations n	136	81	92	77
Mean exacerbations per patient n	0.88	0.53	0.61	0.49
≥1 exacerbation n (%)	72 (46)	50 (33)	62 (41)	47 (30)
≥1 additional exacerbation n (%)	44 (28)	34 (22)	38 (25)	32 (21)
≥2 additional exacerbations n (%)	18 (12)	9 (6)	10 (7)	8 (5)
≥3 additional exacerbations n (%)	10 (6)	4 (3)	4 (3)	3 (2)

Excludes 33 exacerbations (4% of all exacerbations: 31 occurred more than 28 days after the most recent dose; 2 occurred on the day of the first dose).

Moreover, similar mean eDiary-assessed peak expiratory flow, mean rescue medication use, and mean symptom scores were observed during the first and second 2 weeks of dosing periods 1 and 2 (averaged across all 4-weekly dosing periods) across the treatment groups (tables 2–4). Across all treatment groups**,** very few patients (≤2%) had a decrease in peak expiratory flow of ≥20 L·min^−1^, increase of ≥1 or ≥2 occasions per day of rescue medication use or an increase of ≥0.5 or ≥1 in symptom score during the second 2 weeks of a dosing period *versus* the first 2 weeks (tables 2–4). When data from individual 4-weekly dosing periods across the 52-week study period were assessed, there were also no obvious differences in any of these end-points during the first 2 weeks or second 2 weeks of a dosing period (supplementary tables 2–4 and figures 1–3). Moreover, no dose–response relationship was observed for any of the clinical efficacy end-points tested.

**TABLE 2:** Mean eDiary-assessed peak expiratory flow (L·min^−1^) during dosing periods (averaged across all 4-weekly dosing periods within the DREAM study)

**Dosing period**	**Placebo (n=155)**	**Mepolizumab**		
		**75 mg (n=153)**	**250 mg (n=152)**	**750 mg (n=156)**
**First 2 weeks (days 1–14)**				
n^#^	152	151	151	154
Mean (sd)	276.2 (106.3)	278.1 (111.5)	288.4 (125.8)	283.2 (119.8)
**Second 2 weeks (days 15–28)**				
n^#^	152	151	151	154
Mean (sd)	276.6 (108.1)	277.6 (111.3)	286.3 (126.4)	283.1 (120.2)
Mean change (sd)	0.4 (15.2)	−0.5 (7.8)	−2.1 (7.5)	−0.2 (7.5)
≥0 L·min^−1^ decrease	87 (57%)	83 (55%)	99 (66%)	79 (51%)
≥10 L·min^−1^ decrease	14 (9%)	14 (9%)	16 (11%)	9 (6%)
≥20 L·min^−1^ decrease	1 (<1%)	0	3 (2%)	2 (1%)

^#^: Patients with ≥7 days eDiary data for both 2-week periods of at least one dosing interval were included in the analysis.

**TABLE 3:** Mean eDiary-assessed rescue medication use^#^ during dosing periods (averaged across all 4-weekly dosing periods within the DREAM study)

**Dosing period**	**Placebo (n=155)**	**Mepolizumab**		
		**75 mg (n=153)**	**250 mg (n=152)**	**750 mg (n=156)**
**First 2 weeks (days 1–14)**				
n^¶^	152	151	151	154
Mean (sd)	2.0 (2.6)	1.7 (2.8)	2.0 (2.6)	2.0 (2.7)
**Second 2 weeks (days 15–28)**				
n^¶^	152	151	151	154
Mean (sd)	2.1 (2.7)	1.7 (2.9)	2.0 (2.6)	2.0 (2.8)
Mean change (sd)	0.0 (0.3)	−0.0 (0.3)	−0.0 (0.3)	0.0 (0.3)
≥0 occasions per day increase	73 (48%)	68 (45%)	70 (46%)	75 (49%)
≥1 occasion per day increase	1 (<1%)	2 (1%)	1 (<1%)	2 (1%)
≥2 occasions per day increase	0	0	0	1 (<1%)

^#^: Daily rescue medication (short-acting β_2_-agonist) use included salbutamol and albuterol and was defined as occasions per day; ^¶^: patients with ≥7 days of eDiary data for both 2-week periods of at least one dosing interval were included in the analysis.

**TABLE 4:** Mean eDiary-assessed symptom scores during dosing periods (averaged across all 4-weekly dosing periods within the DREAM study)

**Dosing period**	**Placebo (n=155)**	**Mepolizumab**		
		**75 mg (n=153)**	**250 mg (n=152)**	**750 mg (n=156)**
First 2 weeks (days 1–14)				
n^#^	152	151	151	154
Mean (sd)	1.4 (1.1)	1.2 (1.1)	1.4 (1.1)	1.2 (1.1)
Second 2 weeks (days 15–28)				
n^#^	152	151	151	154
Mean (sd)	1.4 (1.1)	1.2 (1.1)	1.3 (1.1)	1.2 (1.1)
Mean change (sd)	−0.0 (0.2)	−0.0 (0.1)	−0.0 (0.2)	0.0 (0.2)
≥0-point increase	77 (51%)	76 (50%)	83 (55%)	75 (49%)
≥0.5-point increase	1 (<1%)	0	1 (<1%)	2 (1%)
≥1-point increase	0	0	1 (<1%)	1 (<1%)

^#^: Patients with ≥7 days of eDiary data for both 2-week periods of at least one dosing interval were included in the analysis.

## Discussion

In this *post hoc* analysis of data from the DREAM study, we found that the response to mepolizumab treatment, as measured by the proportion of patients experiencing exacerbations, as well as changes in mean peak expiratory flow, mean rescue medication use and mean symptom score was similar during the first 2 weeks and second 2 weeks after mepolizumab administration, averaged across all dosing intervals of the 52-week treatment period. Similar results were observed when eDiary data on peak expiratory flow, rescue medication use and symptom score for all 4-weekly dosing intervals were assessed for each individual dosing interval over the 52-week period. In addition, the lowest dose of mepolizumab (75 mg *i.v*.) showed a comparable efficacy profile to the higher doses (250 mg and 750 mg *i.v*.), indicating that no dose–response/dose–period relationship was observed for any of the clinical efficacy end-points. There was also no evidence that baseline blood eosinophil count had any impact on the proportion of patients experiencing exacerbations during dosing periods. Taken together, these data demonstrate for the first time that the response to mepolizumab is sustained over the dosing periods with no differences observed across a ten-fold dose range and across a range of baseline blood eosinophil counts. This suggests that therapeutic benefit is maintained for each mepolizumab dose following long-term treatment.

The long-term efficacy of mepolizumab (up to 4.5 years) has been demonstrated in the COLUMBA study [12]. However, none of the previous clinical studies of mepolizumab have evaluated the durability of the therapeutic response during dosing periods, and in particular, whether there is a reduced response between mepolizumab injections. In our analysis we found that for all the parameters studied, similar results were observed in the first and second 2 weeks of the 4-weekly dosing period. Only a few patients showed a decrease in peak expiratory flow of ≥20 L·min^−1^, an increase of ≥1 occasions per day in rescue medication use or an increase in symptom score ≥0.5 during the second 2 weeks compared with the first 2 weeks of treatment, and the results were similar across the placebo and mepolizumab treatment groups. There are several reasons why patients may experience a loss of treatment efficacy over time including suboptimal dosing, low serum drug levels, immunogenicity (anti-drug and neutralising antibodies), intermittent or episodic therapy, or low treatment adherence [13]. However, our results indicate that the dose intervals for mepolizumab are appropriate, given that the majority of the patients included in this analysis had a sustained treatment response to mepolizumab during the 4-weekly dosing period across the 52-week study.

As expected, the number of exacerbations experienced by patients receiving mepolizumab was reduced compared with those patients receiving placebo, consistent with the findings from previous studies [7–10]. In addition, when evaluating the proportion of patients experiencing additional exacerbations in the second 2 weeks compared with the first, a greater proportion of patients had more exacerbations in the placebo group *versus* the treatment groups. However, the other parameters assessed in this study showed less of a clinical response compared with placebo, potentially indicating that they may not be the most appropriate measures for assessing mepolizumab treatment response. In particular, we did not observe any marked differences in symptom score between the mepolizumab treatment groups and placebo in the first or second 2 weeks after treatment. This could be explained by the entry criteria for this study, as patients were not required to have inadequate asthma symptom control (based on an Asthma Control Questionnaire (ACQ) score of ≥1.5), and therefore patients with good asthma control but with a history of exacerbations would have been eligible to participate in DREAM.

The low symptom scores observed in this study hampered our ability to detect potential treatment differences and may be the result of several factors. First, current measures for assessing asthma symptoms were developed and validated in patients with uncontrolled asthma, but not those with severe disease. Therefore, these measures may not accurately reflect the spectrum of symptoms in all patients with asthma. It is also possible that symptom score may not be a sensitive enough measure to assess control in severe asthma, especially given that baseline symptom scores were low and scores across the treatment period remained low. Alternatively, rescue courses of OCSs may have reduced symptoms more often for patients receiving placebo compared with those receiving mepolizumab. Other tools used in clinical studies include the ACQ score, which has also been used to demonstrate improvements from baseline in asthma control with mepolizumab treatment [7, 8]. In addition, other studies have used St George's Respiratory Questionnaire (SGRQ) total scores to assess the impact of treatment on a patient's quality of life. For example, in the MENSA and MUSCA studies, significant improvements from baseline in SGRQ total score were observed in patients receiving mepolizumab compared with those receiving placebo, and these improvements were particularly evident within the SGRQ symptom domain [7, 8]. Therefore, in the future it may be necessary to use more sensitive instruments, such as SGRQ or ACQ, to more definitively show that there is a sustained effect over the 4-weekly dosing period in patients receiving mepolizumab. However, it is important to note that these measures may not be suitable for daily use.

Patients with severe eosinophilic asthma receiving mepolizumab or placebo in the DREAM study could continue taking daily rescue medication as needed to relieve any asthma symptoms. There were very few differences in rescue medication use in the first 2 weeks after mepolizumab administration compared with the second 2 weeks when assessing the data from the individual 4-weekly dosing periods, and the averaged data over the 52 weeks followed a similar trend. This may reflect the low symptom scores seen in this study, as patients may not need to use rescue medication as frequently if they are experiencing only a low level of asthma symptoms.

Mepolizumab is associated with reductions in blood eosinophil counts in patients with severe eosinophilic asthma [8–10], and activated eosinophils are thought to be responsible for airway remodelling and tissue damage in patients with asthma, potentially leading to a decline in lung function and the development of fixed airway obstruction [14]. Data from the DREAM study showed that blood eosinophil counts were reduced in a dose-dependent manner [9], and with minimal differences seen between doses. In addition, a sub-analysis of data from the same study demonstrated that mepolizumab was associated with a more pronounced dose-dependent reduction in sputum eosinophil counts [15]. No information on eosinophil activation levels was collected during DREAM, and although blood eosinophil counts and the biomarker exhaled nitric oxide fraction were evaluated during the study, they were assessed no more than every 4 weeks. Therefore, the durability of the response to mepolizumab based on eosinophil levels, activation status and airway eosinophilia remains to be determined.

There are several limitations that should be taken into account when considering the results from this analysis. First, the DREAM study was a randomised clinical trial, and therefore the data analysed here were not based on information collected during routine clinical practice. Patients participating in the DREAM study were likely to have severe disease and to have been fully adherent to study treatment and to background anti-asthma controller medications; as a result, our findings may not be fully representative of the experience of the general severe asthma population in the real world. Second, during this study patients received *i.v*. mepolizumab, whereas currently mepolizumab is approved for *s.c*. delivery. However, 75 mg *i.v.* has been shown to be bioequivalent to 100 mg *s.c*. [11], therefore it is likely that a similar pattern in terms of response would be expected with *s.c*. administration. Third, the collection of data on peak expiratory flow, rescue medication use, and symptom score relied on patients completing their eDiary accurately and in a timely manner. In addition, treatment differences *versus* placebo were relatively small for eDiary data and in particular, responses for symptom scores were low, making it difficult to measure the durability of the response based on these end-points. It is also possible that a small number of patients who withdrew from the study may have affected our results as any subsequent eDiary data would not have been captured. In addition, in this analysis we averaged data across all 4-weekly dosing intervals, which assumes that there was the same underlying effect of mepolizumab each month, and this appears to be a reasonable assumption given that similar results were observed when evaluating the individual dosing periods. While we were able to investigate peak expiratory flow, rescue medication use, and symptom score in all individual 4-weekly dosing intervals, only a relatively small number of exacerbations occurred within each interval and so data had to be combined across dosing periods to assess the change in this end-point during the first and second 2 weeks after dosing. Furthermore, as patients with severe eosinophilic asthma are likely to receive long-term mepolizumab treatment [16], additional studies are required to determine whether the response to mepolizumab is sustained during dosing periods when treatment is extended beyond 1 year. Given the heterogenous nature of asthma, it will also be important for future studies to assess whether there is variation in the durability of the mepolizumab response in different subgroups of patients.

In conclusion, this analysis demonstrates that the response to mepolizumab in the first 2 weeks after treatment was similar to that observed in the second 2 weeks. This trend was observed across all doses, and overall our findings indicate that the current dosing regimen used for mepolizumab treatment is appropriate for patients with severe eosinophilic asthma.

## Supplementary material

10.1183/23120541.00068-2020.Supp1 **Please note:** supplementary material is not edited by the Editorial Office, and is uploaded as it has been supplied by the author.
Supplementary material 00068-2020.supplement

00068-2020.supplement.pdf
