# Supplementary material for: Response to mepolizumab treatment is sustained across 4-weekly dosing periods
Source: ERJ Open Res. 2020 Sep 14;6(3):00068-2020. doi: 10.1183/23120541.00068-2020 (PMC7487353; doi:10.1183/23120541.00068-2020)

Supplementary Materials

Table S1. Number of exacerbations during dosing periods (averaged across all 4-weekly dosing periods within the DREAM study) according to baseline blood eosinophil count.

Baseline blood eosinophil count	Dosing period	Placebo (n=155)	Mepolizumab 75 mg (n=153)	Mepolizumab 250 mg (n=152)	Mepolizumab 750 mg (n=156)
<300 cells/µL	**First 2 weeks (Days 1–14)**				
	n	69	84	82	79
	Total exacerbations, n	46	45	59	30
	Mean no. of exacerbations per patient	0.67	0.54	0.72	0.38
	≥1 exacerbation, n (%)	29 (42)	28 (33)	32 (39)	20 (25)
	**Second 2 weeks (Days 15–28)**				
	n	69	84	82	79
	Total exacerbations, n	37	47	62	32
	Mean no. of exacerbations per patient	0.54	0.56	0.76	0.41
	≥1 exacerbation, n (%)	25 (36)	27 (32)	35 (43)	23 (29)
	≥1 additional exacerbation	15 (22)	18 (21)	24 (29)	17 (22)
	≥2 additional exacerbations	4 (6)	5 (6)	9 (11)	2 (3)
	≥3 additional exacerbations	2 (3)	3 (4)	4 (5)	1 (1)
≥300 cells/µL	**First 2 weeks (Days 1–14)**				
	n	86	69	70	77
	Total exacerbations, n	91	22	25	39
	Mean no. of exacerbations per patient	1.06	0.32	0.36	0.51
	≥1 exacerbation, n (%)	46 (53)	16 (23)	20 (29)	26 (34)
	**Second 2 weeks (Days 15–28)**				
	n	86	69	70	77
	Total exacerbations, n	99	34	30	45
	Mean no. of exacerbations per patient	1.15	0.49	0.43	0.58
	≥1 exacerbation, n (%)	47 (55)	23 (33)	27 (39)	24 (31)
	≥1 additional exacerbation	29 (34)	16 (23)	14 (20)	15 (19)
	≥2 additional exacerbations	14 (16)	4 (6)	1 (1)	6 (8)
	≥3 additional exacerbations	8 (9)	1 (1)	0	2 (3)

Excludes 33 exacerbations (4% of all exacerbations: 31 occurred more than 28 days after the most recent dose; 2 occurred on the day of the first dose).

Table S2. Mean eDiary-assessed peak expiratory flow (L/min) during the individual dosing periods of the DREAM study

Dosing period	Placebo (n=155)	Mepolizumab 75 mg (n=153)	Mepolizumab 250 mg (n=152)	Mepolizumab 750 mg (n=156)
**Weeks 1–4**				
n	146	141	149	146
**First 2 weeks**				
Mean (SD)	270.1 (102.2)	271.6 (112.2)	287.3 (120.8)	278.8 (120.3)
**Next 2 weeks**				
Mean (SD)	273.7 (100.4)	280.6 (114.1)	286.0 (123.2)	282.4 (122.4)
Mean change (SD)	3.5 (35.9)	9.0 (28.1)	-1.3 (32.7)	3.6 (27.9)
**Weeks 5–8**				
n	139	140	141	145
**First 2 weeks**				
Mean (SD)	276.0 (106.8)	280.1 (118.2)	293.1 (126.7)	285.8 (121.9)
**Next 2 weeks**				
Mean (SD)	278.4 (113.5)	282.3 (119.4)	289.5 (125.7)	284.8 (120.7)
Mean change (SD)	2.4 (27.9)	2.2 (25.8)	-3.6 (25.1)	-1.1 (26.4)
**Weeks 9–12**				
n	137	134	135	137
**First 2 weeks**				
Mean (SD)	276.3 (111.2)	282.1 (116.6)	305.7 (129.3)	289.4 (124.2)
**Next 2 weeks**				
Mean (SD)	272.0 (111.0)	282.3 (117.3)	299.6 (124.6)	287.8 (123.7)
Mean change (SD)	-4.3 (32.8)	0.2 (25.7)	-6.1 (23.5)	-1.6 (24.4)
**Weeks 13–16**				
n	133	131	135	138
**First 2 weeks**				
Mean (SD)	275.7 (116.0)	280.2 (120.2)	288.5 (124.4)	286.9 (124.3)
**Next 2 weeks**				
Mean (SD)	274.4 (117.1)	276.3 (118.8)	282.8 (124.2)	285.4 (126.7)
Mean change (SD)	-1.3 (24.2)	-3.9 (20.4)	-5.7 (25.2)	-1.4 (21.5)
**Weeks 17–20**				
n	131	130	133	134
**First 2 weeks**				
Mean (SD)	278.3 (114.9)	287.2 (113.8)	288.9 (129.4)	284.7 (127.8)
**Next 2 weeks**				
Mean (SD)	279.8 (115.1)	288.3 (119.5)	286.0 (128.0)	283.0 (125.6)
Mean change (SD)	1.5 (32.8)	1.1 (25.9)	-2.8 (22.4)	-1.7 (21.3)
**Weeks 21–24**				
n	118	122	126	134
**First 2 weeks**				
Mean (SD)	289.0 (114.2)	285.4 (122.1)	291.6 (132.1)	283.6 (124.1)
**Next 2 weeks**				
Mean (SD)	286.1 (114.8)	279.0 (119.8)	288.3 (131.3)	285.6 (126.9)
Mean change (SD)	-2.9 (24.5)	-6.4 (21.2)	-3.3 (19.2)	2.0 (20.8)
**Weeks 25–28**				
n	129	122	127	126

**First 2 weeks**				
Mean (SD)	283.1 (113.8)	280.1 (121.7)	290.0 (128.3)	282.4 (124.8)
**Next 2 weeks**				
Mean (SD)	281.0 (113.8)	278.2 (118.1)	291.6 (128.0)	277.2 (121.8)
Mean change (SD)	-2.0 (23.6)	-1.9 (26.7)	1.6 (18.6)	-5.2 (22.5)
**Weeks 29–32**				
n	123	123	129	127
**First 2 weeks**				
Mean (SD)	283.4 (111.0)	270.5 (109.3)	296.1 (130.9)	283.7 (124.5)
**Next 2 weeks**				
Mean (SD)	282.8 (114.4)	266.5 (103.4)	296.2 (134.8)	283.6 (123.8)
Mean change (SD)	-0.6 (29.8)	-4.0 (22.4)	0.1 (18.9)	-0.1 (20.1)
**Weeks 33–36**				
n	117	121	128	120
**First 2 weeks**				
Mean (SD)	289.8 (114.5)	269.8 (115.3)	296.8 (134.3)	288.1 (125.0)
**Next 2 weeks**				
Mean (SD)	287.7 (114.7)	269.3 (113.4)	296.1 (134.7)	289.3 (128.7)
Mean change (SD)	-2.1 (24.4)	-0.4 (17.0)	-0.7 (18.0)	1.2 (20.1)
**Weeks 37–40**				
n	118	120	121	121
**First 2 weeks**				
Mean (SD)	287.9 (111.9)	274.7 (117.4)	300.4 (131.3)	287.6 (126.9)
**Next 2 weeks**				
Mean (SD)	284.4 (111.9)	272.8 (116.7)	299.2 (131.7)	288.9 (128.0)
Mean change (SD)	-3.5 (27.6)	-1.9 (20.8)	-1.2 (22.4)	1.3 (25.9)
**Weeks 41–44**				
n	118	120	123	121
**First 2 weeks**				
Mean (SD)	283.7 (112.5)	275.3 (114.5)	295.7 (127.5)	287.1 (129.9)
**Next 2 weeks**				
Mean (SD)	284.0 (111.4)	272.3 (115.8)	293.5 (131.0)	288.3 (128.8)
Mean change (SD)	0.2 (21.1)	-2.9 (23.6)	-2.2 (19.6)	1.2 (21.3)
**Weeks 45–48**				
n	115	109	126	117
**First 2 weeks**				
Mean (SD)	280.2 (107.7)	276.2 (110.9)	298.7 (134.4)	287.7 (124.3)
**Next 2 weeks**				
Mean (SD)	278.6 (107.6)	275.0 (110.5)	297.5 (133.4)	288.5 (126.8)
Mean change (SD)	-1.6 (24.6)	-1.2 (21.1)	-1.2 (21.4)	0.8 (31.7)
**Weeks 49–52**				
n	106	113	113	111
**First 2 weeks**				
Mean (SD)	285.1 (113.8)	278.0 (114.9)	294.0 (130.3)	293.3 (128.1)
**Next 2 weeks**				
Mean (SD)	283.5 (117.6)	276.1 (111.6)	293.4 (134.1)	293.3 (129.7)
Mean change (SD)	-1.5 (22.7)	-1.9 (22.1)	-0.7 (15.5)	0.0 (21.4)

Patients with ≥7 days eDiary data for both 2-week periods were included in the analysis of each individual dosing period.
SD, standard deviation.

Table S3. Mean eDiary-assessed rescue medication use* during the individual dosing periods of the DREAM study

Dosing period	Placebo (n=155)	Mepolizumab 75 mg (n=153)	Mepolizumab 250 mg (n=152)	Mepolizumab 750 mg (n=156)
**Weeks 1–4**				
n	147	142	149	147
**First 2 weeks**				
Mean (SD)	2.4 (2.5)	2.1 (2.6)	2.3 (2.6)	2.3 (2.5)
**Next 2 weeks**				
Mean (SD)	2.1 (2.5)	1.9 (2.6)	2.2 (2.7)	2.2 (2.7)
Mean change (SD)	-0.4 (1.0)	-0.2 (0.7)	-0.1 (1.0)	-0.1 (0.8)
**Weeks 5–8**				
n	142	141	142	145
**First 2 weeks**				
Mean (SD)	2.0 (2.3)	1.7 (2.1)	1.9 (2.4)	2.0 (2.6)
**Next 2 weeks**				
Mean (SD)	2.1 (2.5)	1.6 (2.4)	2.0 (2.5)	2.1 (2.8)
Mean change (SD)	0.1 (0.8)	-0.1 (0.7)	0.0 (0.6)	0.0 (0.8)
**Weeks 9–12**				
n	137	135	136	137
**First 2 weeks**				
Mean (SD)	2.0 (2.6)	1.5 (2.4)	1.9 (2.6)	1.9 (2.8)
**Next 2 weeks**				
Mean (SD)	2.0 (3.0)	1.5 (2.4)	1.9 (2.5)	1.9 (2.9)
Mean change (SD)	0.1 (0.9)	-0.0 (0.5)	-0.0 (0.6)	0.0 (0.7)
**Weeks 13–16**				
n	133	133	135	138
**First 2 weeks**				
Mean (SD)	2.0 (2.9)	1.6 (2.5)	2.0 (2.5)	2.0 (2.9)
**Next 2 weeks**				
Mean (SD)	2.0 (2.8)	1.6 (2.6)	2.0 (2.6)	2.0 (3.0)
Mean change (SD)	0.0 (0.7)	0.0 (0.4)	0.1 (0.6)	0.0 (0.4)
**Weeks 17–20**				
n	132	130	133	134
**First 2 weeks**				
Mean (SD)	2.0 (3.0)	1.5 (2.5)	1.9 (2.6)	1.9 (2.8)
**Next 2 weeks**				
Mean (SD)	1.9 (2.9)	1.5 (2.9)	1.8 (2.5)	1.9 (2.8)
Mean change (SD)	-0.1 (0.8)	0.0 (1.1)	-0.1 (0.6)	-0.0 (0.6)
**Weeks 21–24**				
n	118	122	126	134
**First 2 weeks**				
Mean (SD)	1.9 (2.7)	1.6 (3.0)	2.0 (2.6)	2.0 (2.9)
**Next 2 weeks**				
Mean (SD)	2.0 (2.9)	1.6 (3.0)	1.9 (2.7)	2.0 (3.0)
Mean change (SD)	0.1 (0.7)	-0.0 (0.4)	-0.0 (0.5)	0.1 (0.7)
**Weeks 25–28**				
n	130	122	127	126
**First 2 weeks**				

Mean (SD)	2.0 (2.8)	1.6 (3.0)	1.8 (2.6)	2.0 (3.0)
**Next 2 weeks**				
Mean (SD)	2.0 (2.8)	1.6 (3.0)	1.9 (2.7)	2.1 (3.1)
Mean change (SD)	-0.0 (0.5)	0.0 (0.3)	0.1 (0.5)	0.1 (0.6)
**Weeks 29–32**				
n	124	123	129	127
**First 2 weeks**				
Mean (SD)	1.9 (2.8)	1.7 (3.1)	1.9 (2.7)	2.0 (2.7)
**Next 2 weeks**				
Mean (SD)	2.0 (3.0)	1.7 (3.1)	1.9 (2.7)	2.0 (3.1)
Mean change (SD)	0.0 (0.8)	-0.0 (0.4)	0.0 (0.4)	-0.0 (1.0)
**Weeks 33–36**				
n	120	121	128	120
**First 2 weeks**				
Mean (SD)	2.0 (3.0)	1.6 (3.0)	1.9 (2.7)	2.0 (3.0)
**Next 2 weeks**				
Mean (SD)	2.1 (3.2)	1.7 (3.1)	1.8 (2.6)	2.0 (3.1)
Mean change (SD)	0.2 (1.0)	0.0 (0.5)	-0.1 (0.6)	0.0 (0.4)
**Weeks 37–40**				
n	119	120	122	121
**First 2 weeks**				
Mean (SD)	1.9 (3.0)	1.7 (3.1)	1.7 (2.6)	1.9 (3.1)
**Next 2 weeks**				
Mean (SD)	2.0 (3.0)	1.7 (3.0)	1.7 (2.6)	2.0 (3.1)
Mean change (SD)	0.1 (0.7)	-0.0 (0.5)	0.0 (0.6)	0.1 (0.5)
**Weeks 41–44**				
n	119	120	124	121
**First 2 weeks**				
Mean (SD)	2.0 (3.1)	1.6 (3.1)	1.7 (2.5)	1.9 (2.7)
**Next 2 weeks**				
Mean (SD)	2.1 (3.2)	1.6 (3.0)	1.7 (2.5)	1.9 (2.7)
Mean change (SD)	0.1 (0.6)	0.0 (0.6)	-0.0 (0.5)	-0.0 (0.5)
**Weeks 45–48**				
n	116	109	126	117
**First 2 weeks**				
Mean (SD)	1.9 (2.7)	1.7 (3.1)	1.8 (2.6)	2.0 (2.7)
**Next 2 weeks**				
Mean (SD)	2.0 (2.9)	1.7 (3.1)	1.8 (2.7)	1.9 (2.5)
Mean change (SD)	0.1 (0.6)	0.0 (0.4)	0.1 (0.4)	-0.0 (0.6)
**Weeks 49–52**				
n	106	113	113	111
**First 2 weeks**				
Mean (SD)	2.1 (3.2)	1.6 (3.0)	1.8 (2.6)	1.8 (2.5)
**Next 2 weeks**				
Mean (SD)	2.2 (3.4)	1.7 (3.1)	1.8 (2.7)	1.8 (2.7)
Mean change (SD)	0.1 (0.8)	0.1 (0.4)	0.1 (0.4)	0.0 (0.7)

*Daily rescue medication (short-acting β_2-agonist) use included salbutamol and albuterol and was defined as occasions/day. Patients with ≥7 days eDiary data for both 2-week periods were included in the analysis of each individual dosing period.
SD, standard deviation.

Table S4. Mean eDiary-assessed symptom scores during the individual dosing periods of the DREAM study

Dosing period	Placebo (n=155)	Mepolizumab 75 mg (n=153)	250 mg (n=152)	750 mg (n=156)
**Weeks 1–4**				
n	147	142	149	147
**First 2 weeks**				
Mean (SD)	1.7 (1.1)	1.5 (1.1)	1.6 (1.2)	1.5 (1.2)
**Next 2 weeks**				
Mean (SD)	1.5 (1.2)	1.4 (1.2)	1.5 (1.2)	1.3 (1.2)
Mean change (SD)	-0.2 (0.6)	-0.1 (0.5)	-0.1 (0.5)	-0.2 (0.5)
**Weeks 5–8**				
n	142	141	142	145
**First 2 weeks**				
Mean (SD)	1.4 (1.2)	1.3 (1.1)	1.4 (1.2)	1.3 (1.2)
**Next 2 weeks**				
Mean (SD)	1.5 (1.3)	1.3 (1.1)	1.4 (1.2)	1.3 (1.2)
Mean change (SD)	0.0 (0.5)	-0.0 (0.5)	0.0 (0.5)	-0.0 (0.4)
**Weeks 9–12**				
n	137	135	136	137
**First 2 weeks**				
Mean (SD)	1.3 (1.2)	1.2 (1.1)	1.4 (1.2)	1.2 (1.2)
**Next 2 weeks**				
Mean (SD)	1.4 (1.2)	1.2 (1.1)	1.4 (1.2)	1.2 (1.2)
Mean change (SD)	0.1 (0.6)	-0.0 (0.4)	0.0 (0.4)	0.0 (0.4)
**Weeks 13–16**				
n	133	133	135	138
**First 2 weeks**				
Mean (SD)	1.4 (1.2)	1.2 (1.2)	1.4 (1.2)	1.2 (1.2)
**Next 2 weeks**				
Mean (SD)	1.4 (1.2)	1.2 (1.2)	1.5 (1.2)	1.2 (1.2)
Mean change (SD)	0.0 (0.5)	0.0 (0.4)	0.1 (0.4)	0.0 (0.3)
**Weeks 17–20**				
n	132	130	133	134
**First 2 weeks**				
Mean (SD)	1.3 (1.1)	1.1 (1.1)	1.3 (1.2)	1.1 (1.2)
**Next 2 weeks**				
Mean (SD)	1.2 (1.1)	1.1 (1.1)	1.3 (1.2)	1.1 (1.1)
Mean change (SD)	-0.1 (0.6)	0.0 (0.3)	-0.0 (0.3)	0.0 (0.4)
**Weeks 21–24**				
n	118	122	126	134
**First 2 weeks**				
Mean (SD)	1.2 (1.2)	1.0 (1.1)	1.4 (1.2)	1.2 (1.2)
**Next 2 weeks**				
Mean (SD)	1.3 (1.2)	1.1 (1.1)	1.4 (1.2)	1.2 (1.2)
Mean change (SD)	0.0 (0.4)	0.0 (0.3)	0.0 (0.3)	0.0 (0.5)
**Weeks 25–28**				
n	130	122	127	126
**First 2 weeks**				

Mean (SD)	1.3 (1.2)	1.1 (1.1)	1.3 (1.3)	1.2 (1.2)
**Next 2 weeks**				
Mean (SD)	1.3 (1.2)	1.1 (1.1)	1.3 (1.2)	1.2 (1.2)
Mean change (SD)	-0.0 (0.5)	-0.0 (0.3)	-0.0 (0.4)	0.0 (0.3)
**Weeks 29–32**				
n	124	123	129	127
**First 2 weeks**				
Mean (SD)	1.2 (1.1)	1.1 (1.1)	1.3 (1.2)	1.1 (1.1)
**Next 2 weeks**				
Mean (SD)	1.3 (1.1)	1.1 (1.1)	1.3 (1.2)	1.2 (1.2)
Mean change (SD)	0.0 (0.5)	-0.0 (0.3)	0.0 (0.4)	0.1 (0.4)
**Weeks 33–36**				
n	120	121	128	120
**First 2 weeks**				
Mean (SD)	1.2 (1.2)	1.1 (1.1)	1.4 (1.2)	1.2 (1.2)
**Next 2 weeks**				
Mean (SD)	1.3 (1.2)	1.1 (1.1)	1.3 (1.2)	1.2 (1.2)
Mean change (SD)	0.0 (0.4)	-0.0 (0.3)	-0.0 (0.4)	0.0 (0.3)
**Weeks 37–40**				
n	119	120	122	121
**First 2 weeks**				
Mean (SD)	1.3 (1.2)	1.1 (1.1)	1.2 (1.2)	1.2 (1.2)
**Next 2 weeks**				
Mean (SD)	1.3 (1.2)	1.1 (1.1)	1.3 (1.2)	1.2 (1.2)
Mean change (SD)	0.0 (0.5)	0.0 (0.3)	0.1 (0.4)	0.0 (0.4)
**Weeks 41–44**				
n	119	120	124	121
**First 2 weeks**				
Mean (SD)	1.3 (1.2)	1.0 (1.1)	1.3 (1.2)	1.2 (1.2)
**Next 2 weeks**				
Mean (SD)	1.3 (1.2)	1.1 (1.1)	1.3 (1.2)	1.1 (1.2)
Mean change (SD)	0.0 (0.3)	0.0 (0.3)	-0.0 (0.4)	-0.0 (0.3)
**Weeks 45–48**				
n	116	109	126	117
**First 2 weeks**				
Mean (SD)	1.2 (1.2)	1.0 (1.0)	1.3 (1.2)	1.1 (1.2)
**Next 2 weeks**				
Mean (SD)	1.3 (1.2)	1.1 (1.1)	1.3 (1.2)	1.2 (1.2)
Mean change (SD)	0.0 (0.6)	0.0 (0.4)	0.0 (0.4)	0.0 (0.3)
**Weeks 49–52**				
n	106	113	113	111
**First 2 weeks**				
Mean (SD)	1.2 (1.2)	1.0 (1.1)	1.2 (1.2)	1.1 (1.2)
**Next 2 weeks**				
Mean (SD)	1.2 (1.2)	1.1 (1.1)	1.3 (1.2)	1.1 (1.1)
Mean change (SD)	0.0 (0.4)	0.0 (0.3)	0.0 (0.3)	-0.0 (0.4)

Patients with ≥7 days eDiary data for both 2-week periods were included in the analysis of each individual dosing period.
SD, standard deviation.

Figure S1. Mean eDiary-assessed change in peak expiratory flow (L/min) during the second 2 weeks compared with the first 2 weeks during the individual dosing periods in the DREAM study

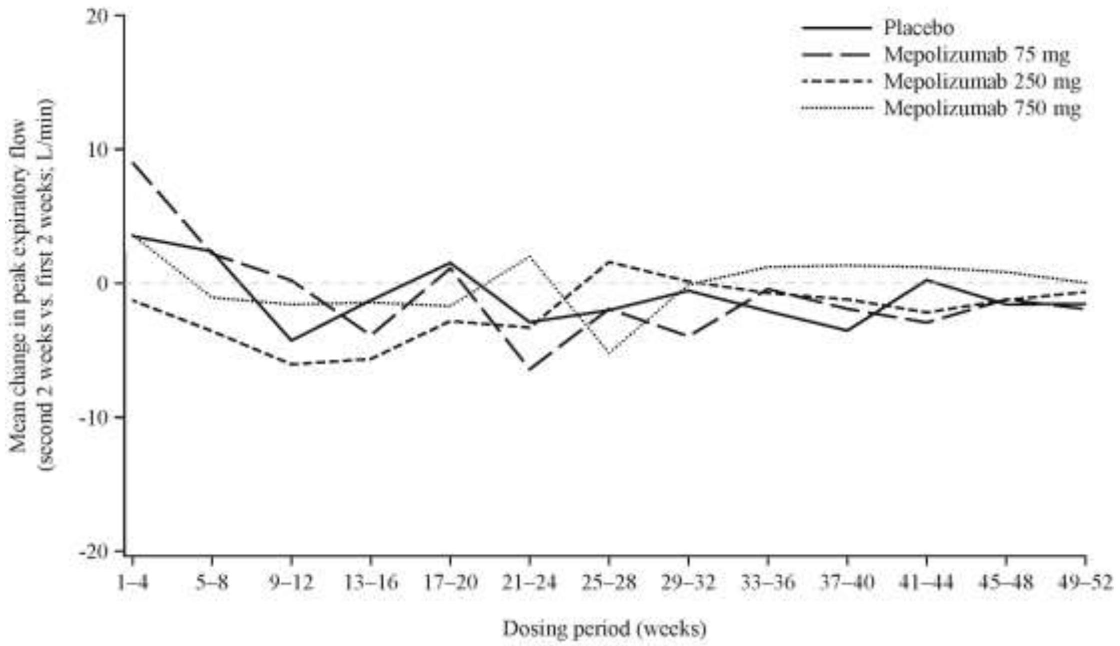

Figure S2. Mean eDiary-assessed change in symptom score during the second 2 weeks compared with the first 2 weeks during the individual dosing periods in the DREAM study

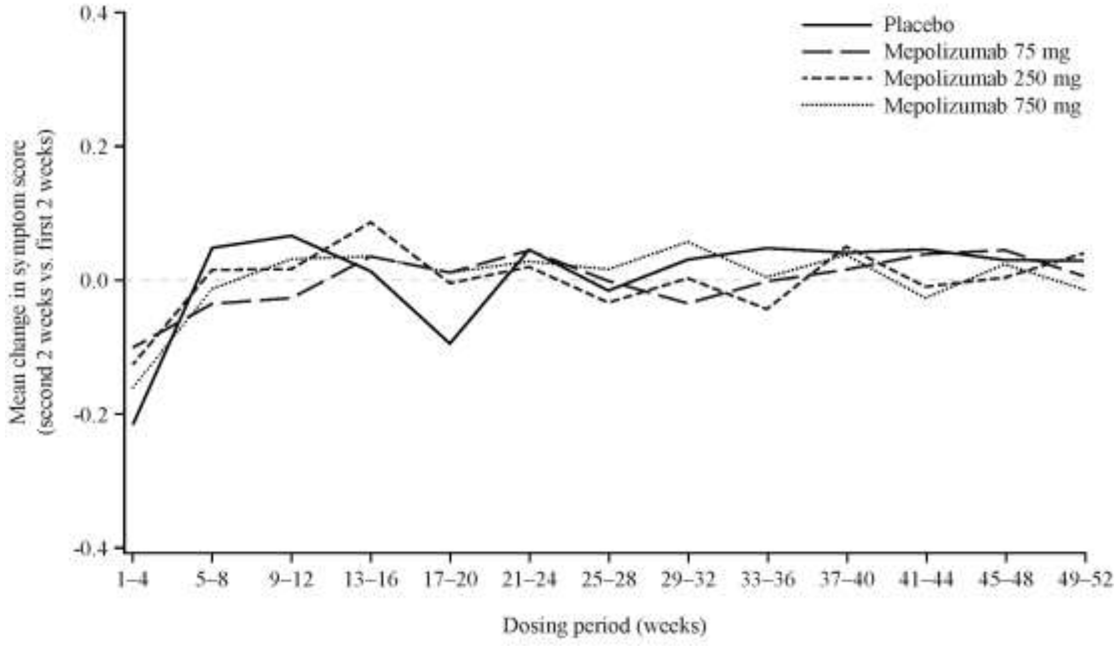

Figure S3. Mean eDiary-assessed change in daily rescue medication use during the second 2 weeks compared with the first 2 weeks during the individual dosing periods in the DREAM study

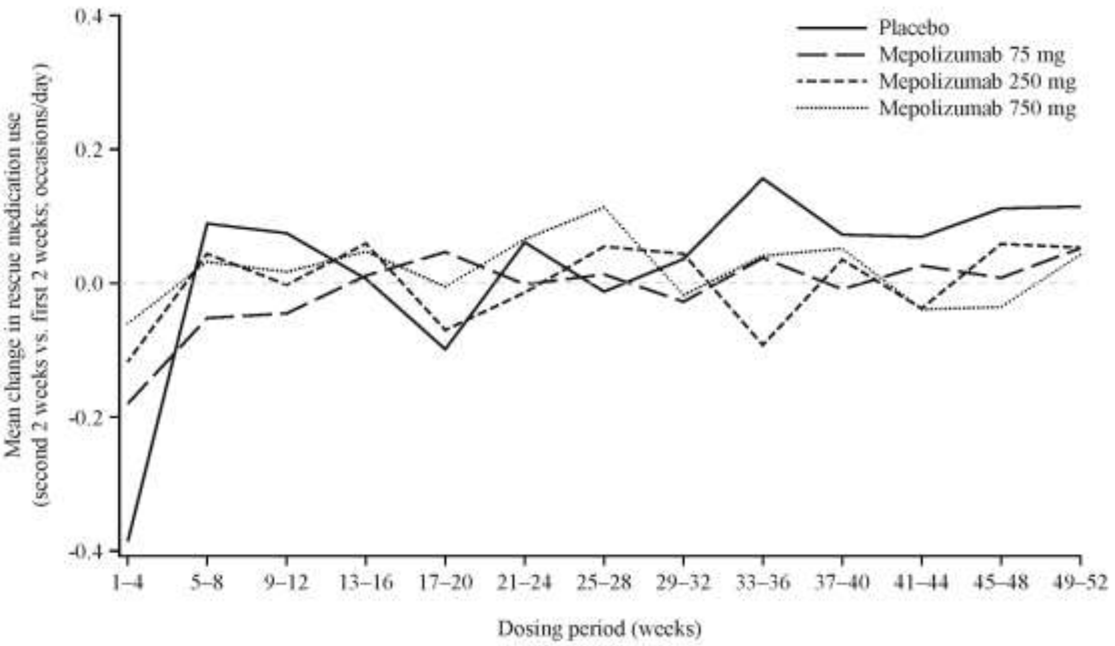

Supplement: Supplementary file 1 [file 00068-2020.supplement.pdf]
